# Terrestrial or marine species distribution model: Why not both? A case study with seabirds

**DOI:** 10.1002/ece3.8272

**Published:** 2021-11-23

**Authors:** Henry Häkkinen, Silviu O. Petrovan, William J. Sutherland, Nathalie Pettorelli

**Affiliations:** ^1^ Institute of Zoology Zoological Society of London London UK; ^2^ Department of Zoology Cambridge University Cambridge UK; ^3^ BioRISC (Biosecurity Research Initiative at St Catharine’s) St Catharine’s College Cambridge UK

**Keywords:** marine, multi‐realm modeling, seabird, seabird ecology, species distribution model, species distribution modeling, terrestrial

## Abstract

Species reliant on both the terrestrial and marine realms present a challenge for conventional species distribution models (SDMs). For such species, standard single‐realm SDMs may omit key information that could result in decreased model accuracy and performance. Existing approaches to habitat suitability modeling typically do not effectively combine information from multiple realms; this methodological gap can ultimately hamper management efforts for groups such as seabirds, seals, and turtles. This study, for the first time, jointly incorporates both terrestrial information and marine information into a single species distribution model framework. We do this by sampling nearby marine conditions for a given terrestrial point and vice versa using parameters set by each species’ mean maximum foraging distance and then use standard SDM methods to generate habitat suitability predictions; therefore, our method does not rely on post hoc combination of several different models. Using three seabird species with very different ecologies, we investigate whether this new multi‐realm approach can improve our ability to identify suitable habitats for these species. Results show that incorporating terrestrial information into marine SDMs, or vice versa, generally improves model performance, sometimes drastically. However, there is considerable variability between species in the level of improvement as well as in the particular method that produces the most improvement. Our approach provides a repeatable and transparent method to combine information from multiple ecological realms in a single SDM framework. Important advantages over existing solutions include the opportunity to, firstly, easily combine terrestrial and marine information for species that forage large distances inland or out to sea and, secondly, consider interactions between terrestrial and marine variables.

## Graphical abstract

Species reliant on both the terrestrial and marine realms present a challenge for conventional species distribution models (SDMs). This study, for the first time, jointly incorporates both terrestrial information and marine information into a single species distribution model framework. Results show that incorporating terrestrial information into marine SDMs, or vice versa, generally improves model performance, sometimes drastically.

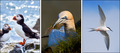

## 1 INTRODUCTION

Species distribution models (SDMs) are a widely used method in ecology to describe, predict, and project species ranges (Engler et al., 2017; Loiseau et al., 2020; Mod et al., 2016; Robinson et al., 2017). In a conventional SDM approach, a species’ occurrence is correlated with local environmental conditions, whether climatic or biotic, to approximate the species’ ecological niche. Such models assume each species’ niche is distinct, has a defined and temporally fixed relationship with environmental factors, and is reasonably consistent across a species range (Elith & Leathwick, 2009; Maguire et al., 2015). However, since these assumptions are not always true, several more sophisticated SDMs have emerged that allow greater flexibility in the way we model species ranges; examples include seasonal niche SDMs (Engler et al., 2014; Nakazawa et al., 2004), multiple life‐stage SDMs (Taboada et al., 2013), and multi‐state SDMs (Frans et al., 2018; Gherghel et al., 2020). This new wave of SDMs targets species that change their environmental requirements at different parts of their lifecycle, such as in anadromous fish or amphibians, or at different times of year, such as in migratory birds. However, for species in which individuals have different environmental preferences in different parts of their range, such as central foraging species, amphibious species that live on both land and freshwater or land and the sea, options on how to model effectively these disparate parts of their ranges are still limited. There are numerous organisms that rely on multiple realms (hereafter referred to as multi‐realm species), such as the marine and terrestrial realm, yet few models effectively acknowledge this dependency (but see Gherghel et al., 2020). This gap may hamper our ability to predict, project, and ultimately manage the conservation of seals, sea turtles, and seabirds, among others, particularly in response to the complex multi‐realm effects driven by climate change.

Currently available options to model the habitat suitability of species that span both the terrestrial and marine realms can be differentiated according to the level of integration of multi‐realm data they offer. Most studies focus on the realm most important to the species considered and use a conventional single‐realm SDM; modeling the pelagic non‐breeding range of seabirds can, for example, be achieved using a purely marine SDM (Engler et al., 2017), given that most pelagic seabirds are expected to exhibit no reliance on the terrestrial realm during their non‐breeding season. However, estimating a seabird's breeding range using only terrestrial factors requires strong evidence that the species range is mostly determined by terrestrial factors (unlikely in birds that forage at sea) or that terrestrial variables can act as strong proxies for relevant marine variables (Araújo et al., 2019; Engler et al., 2017). For example, if coastal sea temperature is known to be an important variable shaping habitat suitability of a given species, then terrestrial temperature may be an appropriate proxy if it closely correlates to coastal sea temperature on a relevant spatial scale. However, adequate proxies are not always available; the deliberate omission of marine or terrestrial variables may moreover lead to potentially important ecological processes not being captured by the model, limiting the reliability of its outputs.

Other studies have proposed to build two separate marine and terrestrial SDMs and then subsequently combine them, similar in logic to a multi‐niche or seasonal niche approach (Engler et al., 2014; Nakazawa et al., 2004). This “joining” process can be done in several ways, such as by summing or averaging suitability for each cell (Frans et al., 2018; Gschweng et al., 2012), a “nearest neighbor” approach (Gherghel et al., 2020; Russell et al., 2015) or a “moving window” approach (Frans et al., 2018). However, there are several disadvantages to such approaches. Firstly, many methods of joining SDMs, such as “moving window” or “nearest neighbor,” are not easily transferable between species. A “moving window” analysis requires a threshold of suitability that can be applied to different SDM outputs, which can be difficult and time‐consuming to estimate (Liu et al., 2013). It also requires setting a maximum window parameter, which can be estimated from movement data if such information exists (Frans et al., 2018). “Moving window” analyses are thus computationally intensive and can be sensitive to these two parameters. “Nearest neighbor” analyses can be used for species that do not move far from coastal areas, but these analyses become problematic when considering species that move and forage over large distances since the condition of the nearest marine area may not be relevant if they can simply forage further out to sea. Furthermore, these analyses cannot incorporate interactions between terrestrial and marine variables on species’ distributions; they also do not facilitate comparison of the relative importance of marine and terrestrial variables in shaping the habitat suitability of a given area. More generally, a species may rely on a combination of marine and terrestrial factors in close proximity (e.g., high marine temperature and low terrestrial temperature), which is difficult to capture when the basis of the modeling approach is two separate SDMs. In addition, different parts of a species range may be constrained by different factors: A species may, for example, be constrained by low marine productivity at one end of their range and by high maximum terrestrial temperatures at the other. Current SDM approaches available to predict habitat suitability for species reliant on more than one realm do not offer a way to combine information to allow this form of comparison.

To address this methodological gap, we developed a novel approach to species distribution modeling for species reliant on more than one ecological realm and highlight its benefits using three seabird species with differing life histories. To do so, we first developed and compared possible approaches for incorporating terrestrial and marine environmental variables into a single model framework. Following this, we assessed the interactions and relative importance of marine and terrestrial variables on seabird distributions during the breeding season.

## 2 MATERIALS AND METHODS

### 2.1 Study area and seabird data

**FIGURE 1:**
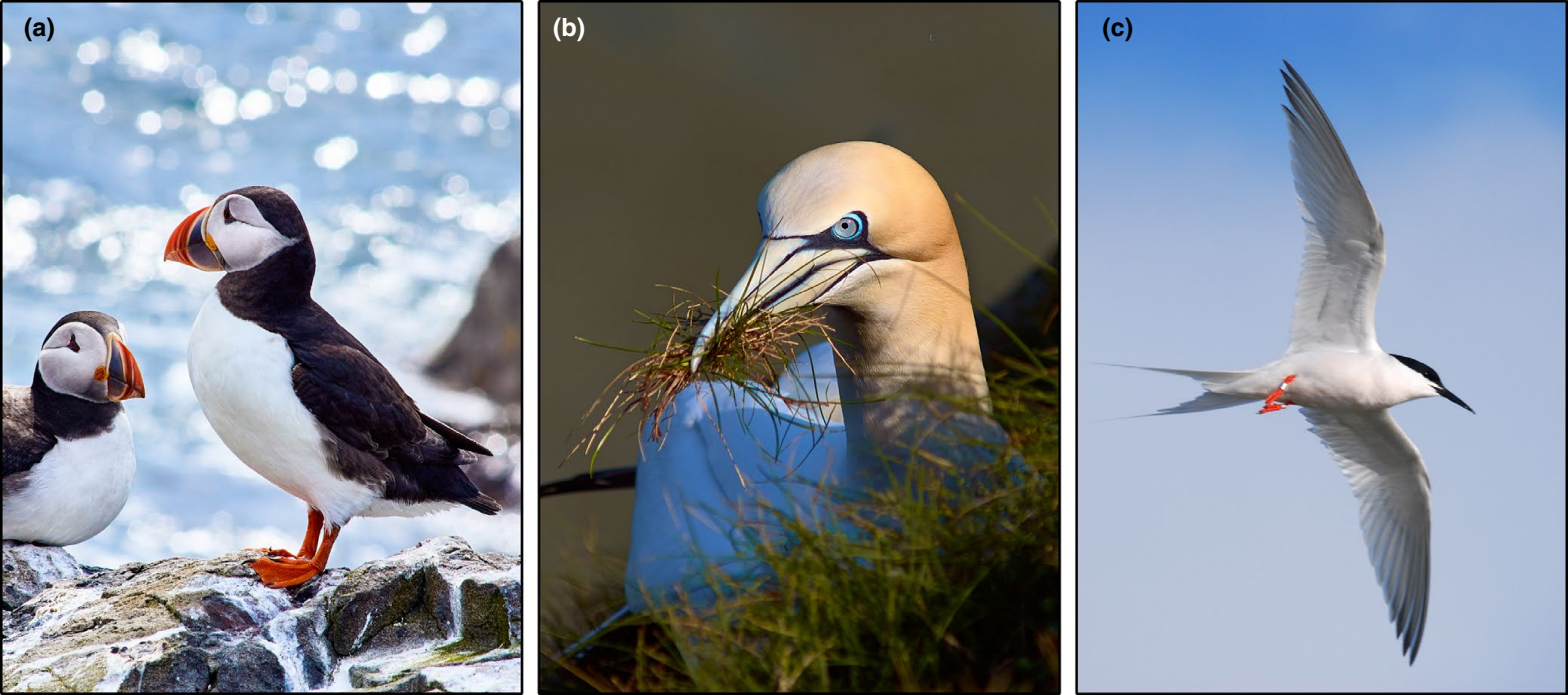
Seabird study species. (a) Atlantic puffin (*Fratercula arctica*), (b) Northern gannet (*Morus bassanus*), (c) Roseate tern (*Sterna dougallii*). (a) and (b) copyright Seppo Häkkinen, (c) copyright Brian Burke from BirdWatch Ireland under the National Parks & Wildlife Service license

We considered the marine and terrestrial breeding range of three European seabird species selected to differ in body sizes, diets, range sizes, migration patterns, and other life‐history traits: Atlantic puffin *Fratercula arctica* (L.), northern gannet *Morus bassanus* (L.), and roseate tern *Sterna dougallii* (Montagu, 1813; Figure 1). These three species enabled us to investigate (1) how robust a multi‐realm SDM approach is across ecologically different species and (2) how different life‐history traits influence the identification of the most important environmental variables for species distribution. The Atlantic puffin is a truly pelagic species during the non‐breeding season but breeds in burrows in soft soil, typically near cliff‐sides, usually with short vegetation and boulder fields (Cramp, 1985; Stanley BirdLife International, 2021a). During the breeding season, puffins typically feed within a few kilometers of their northern European colonies, though occasionally will forage up to 50 km away (BirdLife International, 2021a). Northern gannets are resident across Europe and the Atlantic all year round both near the coast and far out to sea, and typically nest on isolated vertical rocky cliffs (Cramp, 1985; Stanley BirdLife International, 2021b). They undertake foraging trips far from the colony, up to 200–300 km away, and feed by diving into shoals of fish from up to a hundred meters above the sea surface (Garthe et al., 2014; Grecian et al., 2012; Hamer et al., 2001; Thaxter et al., 2012). Roseate terns are partial migrants, but those that breed in Europe migrate long distances from the Southern Hemisphere to occupy a diverse set of breeding habitats across tropical and temperate regions (Cramp, 1985; Stanley BirdLife International, 2021c). They often nest in dense vegetation, including low growing shrubs, or among large rocks and even in burrows (Wilson et al., 2014). During the breeding season, roseate terns feed on or near coastal areas, including in estuaries, though will also sometime forage by plunge diving in deeper water (Wilson et al., 2014).

We aimed to identify the terrestrial and marine areas used by our three species during the breeding season in the northeast Atlantic region as defined by Oslo/Paris convention (OSPAR; https://www.ospar.org/about). Our study area includes any terrestrial area that is within or borders the OSPAR region, with the exceptions of Greenland, Madeira, and the Canaries as they only partially border the OSPAR region. Our study area also includes countries that surround the Baltic Sea, including Finland and the Baltic states and the federal subjects (subregions) of Russia that border the OSPAR region; these adjustments were made in response to known distributions of important fish stocks, as well as areas known to be important breeding and/or wintering grounds for the species considered (Figure S1). We therefore used BirdLife range polygons to generate occurrence data for all species (BirdLife International & Handbook of the Birds of the World, 2020), filtered to include only “present” species and species’ ranges during the breeding season; passage or vagrant ranges were excluded from our analyses. For puffins and roseate tern, this resulted in a defined terrestrial and marine range across the OSPAR region. However, for gannets there is no clear distinction between the range of breeding and non‐breeding populations since the species is resident around the coast of Europe all year round. Therefore, we identified gannets’ breeding sites across Europe (BirdLife International, 2021b) and cropped their marine range to areas within the mean maximum foraging distance (MMFD) of breeding colonies. The MMFD for northern gannets is reported as approximately 200 km (Thaxter et al., 2012) so their effective breeding marine range was defined as any part of their marine range within a 200‐km radius of a breeding colony. Occurrence points were generated from range polygons at a 5‐min spatial resolution. The final occurrence dataset for each species consisted of 58 terrestrial and 90,225 marine points for gannets, 6796 terrestrial and 16,207 marine points for puffins, and 318 terrestrial and 3949 marine points for roseate terns.

**FIGURE 2:**
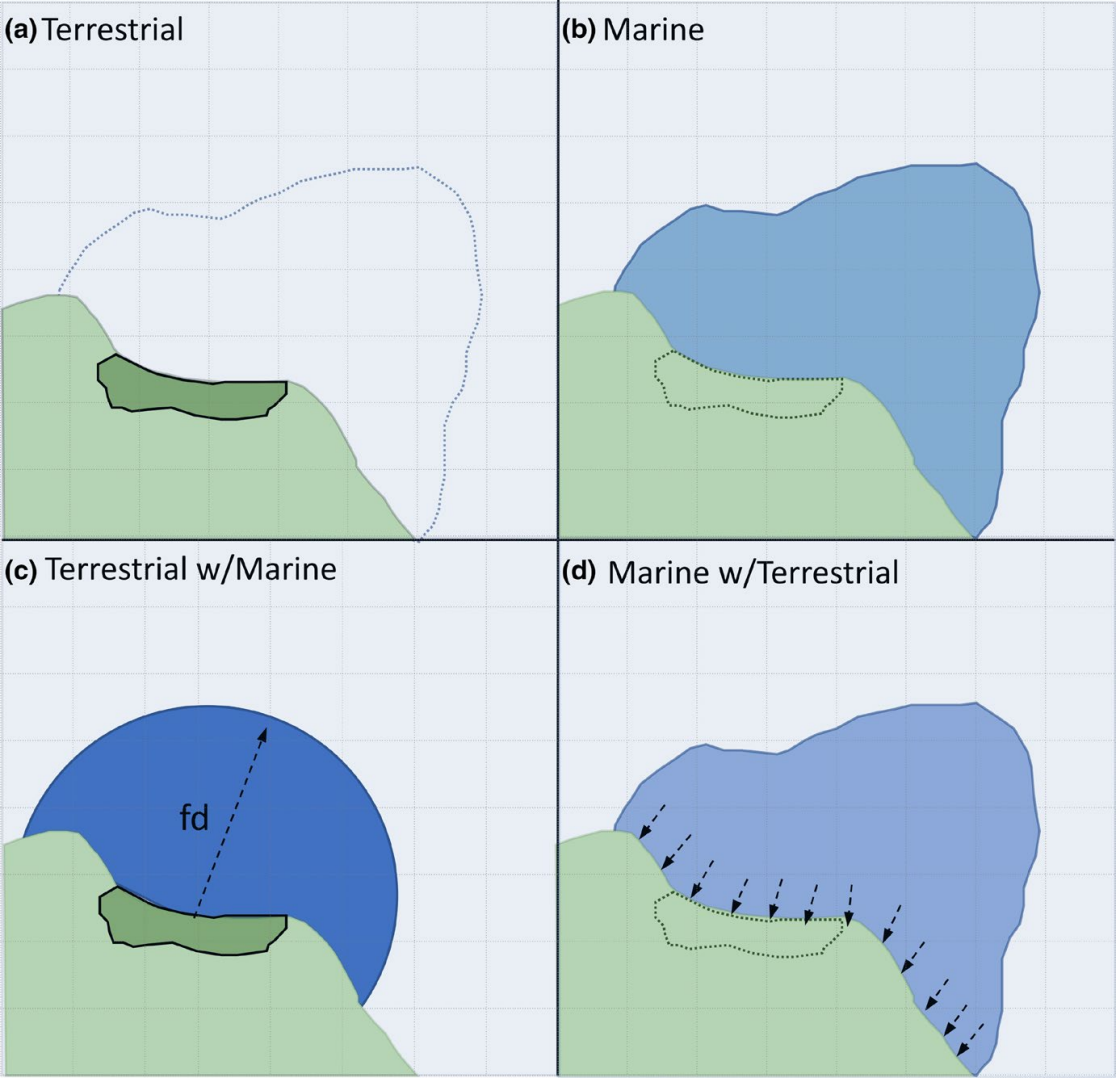
Overview of species distribution model (SDM) single‐ and multi‐realm model types. (a) In a terrestrial‐only SDM, a species range was defined as its terrestrial portion only, and environmental variables were similarly only sampled from terrestrial areas. (b) In a marine‐only SDM, a species range was defined as its marine portion only, and environmental variables were similarly only sampled from marine areas. (c) In a terrestrial with marine components model, all marine points within mean maximum foraging distance (“fd”) of a terrestrial cell are sampled and incorporated into the environmental variable dataset. (d) In a marine with terrestrial components model, for each given marine cell we identify the nearest terrestrial cell and its terrestrial variable values are inherited by the given marine cell

For each species, we generated 10,000 pseudo‐absences, which were randomly drawn from background environmental data outside of occupied grid cells. To ensure the set of pseudo‐absences was not having a significant impact on the model output, we created 5 sets of random pseudo‐absences with 10,000 points each, as well as one smaller set used for diagnostic purposes that contained the same number of pseudo‐absences as the number of presences for the species in question (i.e., prevalence was always 0.5). Since all species breed on, or near, the coast, we restricted terrestrial background data to areas within 20 km of the coastline. Since marine areas outside of foraging range are not available to the species considered, we limited the background marine data to areas within the MMFD from the nearest coastline. For each species, we estimated their mean maximum foraging range from previously published estimates and treated this as a 90^th^ percentile distance. Any marine areas outside of the 100th percentile distance were excluded from the background data. MMFD was defined as 40 km for puffins (Harris et al., 2012), 200 km for gannets (Thaxter et al., 2012), and 16.6 km for roseate terns (Thaxter et al., 2012).

### 2.2 Environmental variables

Previous SDMs for seabirds have identified sea surface temperature (SST), salinity, chlorophyll concentration, bathymetry depth and variance, pH, and sea ice cover as important marine environmental variables shaping seabird distributions (see Engler et al., 2017 for a review). Based on this, and information on the ecology of our chosen species, we decided to include the following marine variables: mean SST during the winter and spring (defined as December to May), mean salinity, maximum surface chlorophyll concentration, bathymetry, and distance to land. Many of these factors do not directly impact seabirds, but act as proxies for marine productivity and prey abundance in a given area; in particular, marine winter conditions are often an effective proxy for prey availability during the summer breeding season (Engler et al., 2017). Monthly SST values were downloaded at 5‐min resolution from the MARSPEC database (Sbrocco & Barber, 2013). Salinity, chlorophyll concentration, and bathymetry data were all downloaded at 5‐min resolution from the BIO‐ORACLE v2.1 database (Assis et al., 2018). If a grid cell had both valid marine and terrestrial values, the distance from land was set to zero; otherwise, distance from land was defined as the distance from each marine grid cell to the nearest grid cell with valid terrestrial values. Distance was estimated in meters using projected data to avoid issues with distortion at high latitudes and the distance function from the raster package (Hijmans, 2021). The final dataset was at 5‐min resolution.

All seabirds nest on land and often show strong spatial and environmental preferences (Engler et al., 2017; Wakefield et al., 2017). Some studies have focussed on the terrestrial preferences of seabirds and have found strong local effects of habitat type, land cover, and vegetation (Engler et al., 2017; Rayner et al., 2007). Seabirds, moreover, display climatic preferences, and a broad range of seabird SDMs have been successfully developed using factors including mean temperature of the warmest month, precipitation during the spring/summer, altitude, and isolation of landmass (Bécares et al., 2015; Russell et al., 2015). Based on these studies, we collated terrestrial environmental variables that are believed to be important in determining distribution ranges for our three species (Bécares et al., 2015; Engler et al., 2017; Russell et al., 2015). The final collated terrestrial variable list was comprised of mean temperature of the warmest month, total precipitation during breeding months (March–August), isolation of the land mass, area of the land mass, land cover, and distance from the sea. Monthly temperature and precipitation variables were downloaded at 5‐min spatial resolution from WorldClim v2.1 (Fick & Hijmans, 2017). Isolation and area of landmass were estimated for each landmass in Europe, for which the base data were downloaded from the Eurostat database (GISCO, 2021). Isolation was defined as the distance from a focal landmass to the nearest larger landmass. Landcover was estimated as the mean and minimum normalized difference vegetation index (NDVI) during the breeding season calculated from monthly data; monthly data were downloaded from the MODIS database at 1‐km resolution (Didan, 2015) and aggregated to our 5‐min resolution. If a grid cell had both valid marine and terrestrial values, the distance from the sea was set to zero; otherwise, distance from the sea was defined as the distance from each terrestrial grid cell to the nearest grid cell with valid marine values. Distance was estimated in meters using projected data to prevent issues with distortion at high latitudes and the distance function from the raster package (Hijmans, 2021). The final dataset was at 5‐min resolution.

### 2.3 Combining marine and terrestrial variables

To combine terrestrial and marine variables in the same model, we needed to relate terrestrial and marine conditions in a meaningful way. We tested two approaches: (1) We considered the mean and standard deviation of marine conditions around a terrestrial point (“Terrestrial with Marine” models), and (2) we considered the nearest terrestrial values to each marine point (“Marine with Terrestrial” models) (Figure 2). In Approach 1, for each terrestrial cell in our study area, we identified the nearest coastline, defined as the nearest cell with valid marine values, and designated this cell as the “nearest coast cell.” We sampled from all marine cells around this nearest coast cell within a radius determined by each species’ MMFD. Sampling was carried out with projected data, to avoid biases associated with calculating distances with latitude/longitude data, using the extract function from the raster package where the buffer was set as the MMFD (Hijmans, 2021). We calculated the mean and standard deviation of all marine variables across the sampled cells and incorporated these into our SDM models. Variables based on the standard deviation were suffixed with the term “variation.” For example, “bathymetry variation” describes the variability of the depth of the ocean floor within a species’ MMFD. Each terrestrial cell therefore had both local terrestrial values and the mean and standard deviation of nearby marine values.

In Approach 2, for each marine cell we identified the nearest terrestrial cell, using the same method as was used to estimate distance from shore. The environmental values associated with this terrestrial cell were added to each marine cell. Each marine cell therefore had both local marine values and the values of the nearest terrestrial cell.

We tested for covariance between pairs of environmental variables using a Pearson correlation. Any two variables with a correlation above .7 were considered highly covarying (Dormann et al., 2013). We found no high covariance between any of the terrestrial factors. Bathymetry and maximum chlorophyll concentration were, however, found to be highly correlated (Pearson's correlation > .7). After combining marine and terrestrial factors, we also found high covariance (Pearson's correlation > .7) between area of nearest land and isolation of nearest land, as well as temperature of the nearest coastline and SST. The final relevant SDMs therefore dropped one of these two paired variables at random, and a second model run was carried out separately containing the other variable. This means in total we ran two additional permutations for marine models (for Bathymetry and maximum chlorophyll concentration) and four permutations for marine with terrestrial component models (area of nearest land/isolation of nearest land, as well as temperature of the nearest coastline/SST). Results are presented for whichever variable of a covarying pair produced a stronger model overall.

### 2.4 Species distribution modeling

In order to estimate parameter values for the collated environmental variables and to assess the relative merit of each of the 4 types of models, we created an ensemble SDM (Araújo & New, 2007). The ensemble model was based on five underlying modeling techniques: generalized linear model (GLM), generalized additive model (GAM), random forest (RF), artificial neural network (ANN), and maximum entropy (MAXENT). Analyses were carried out in R (R Core Team, 2020), using the biomod2 package (Thuiller et al., 2014). We used default SDM settings, with the exception that the maximum number of iterations was increased from 100 to 1000 if convergence did not occur after 100 runs.

We used internal validation to evaluate SDM accuracy. Each dataset was split so that 70% of the presence and pseudo‐absence points were placed in a training dataset to calibrate the models. These models were then used to predict the suitability of the remaining 30% validation data points. We used four metrics to evaluate how accurately the training dataset predicted the validation data: true skill statistic (TSS), the receiver operating curve, sensitivity, and specificity (Lawson et al., 2014). To ensure the composition of the training data set did not affect model accuracy, the process of splitting, calibrating, and validating was repeated five times for each dataset, each time with a different training dataset.

For each of the presence and pseudo‐absence datasets, we made an ensemble forecast for each species across the region of interest. Ensemble models were built using all of our presence and pseudo‐absence points, in order to maximize the information in the model and give the highest level of confidence in parameter values. For each presence and pseudo‐absence dataset, we also created a “full” model to include in the ensemble model. However, to be included in the ensemble a “full” model had to have a TSS of over 0.6. Models with TSS > 0.6 are considered to have “substantial” performance (Landis & Koch, 1977). To make sure our ensemble models were sufficiently similar to, and therefore as robust as, our validation models, we calculated Spearman's correlation between ensemble models constructed from internally validated data and from the full data. A high correlation indicates that the validation and full models are indeed very similar and therefore of a similar accuracy. To construct ensembles, each model (that had a TSS of over 0.6) was rescaled to be on the same numerical scale and then combined to calculate the mean suitability of every grid cell, weighted by the accuracy (TSS) of each model. We also calculated a measure of uncertainty in suitability across the region by estimating the variance in suitability across all models. While there are other methods to estimate mean suitability, mean weighted suitability is a generally robust ensemble method that accounts for model accuracy (Gritti et al., 2013).

The importance of each variable in the SDM was estimated for each model (full and validation alike). For each given environmental variable, the variable was randomized, and a new SDM was generated with the randomized variable. Pearson's correlation (*r*) was then calculated between two models, one made with the true variable values and one with the randomized variable. Variable importance is scaled from 0 to 1, where a value of 0 implies a given variable has almost no impact on the model.

## 3 RESULTS

All models performed well: Validation TSS scores were above 0.6 in all cases. The Spearman correlation between validation and full datasets was >.7 in all cases (Table 1), which indicates that the TSS and ROC scores from internal validation models reflect the accuracy of models calculated with all available data.

**TABLE 1:** Summary statistics for species distribution models (SDMs) for each species and modeling approach

Species	Approach	TSS	ROC	Spearman's *p*
Atlantic puffin	TerrOnly	0.76 (0.02)	0.95 (0.01)	.99 (<.01)
	MarOnly	0.65 (0.03)	0.91 (0.01)	.99 (<.01)
	**Terr w/Mar**	**0.83 (0.02)**	**0.97 (<0.01)**	.**99 (<.01)**
	Mar w/Terr	0.69 (0.03)	0.93 (0.01)	.99 (<.01)
Northern gannet	TerrOnly	0.68 (0.10)	0.87 (0.05)	.74 (.17)
	MarOnly	0.69 (0.04)	0.89 (0.02)	.99 (<.01)
	Terr w/Mar	0.72 (0.13)	0.88 (0.07)	.93 (.02)
	**Mar w/Terr**	**0.75 (0.04)**	**0.93 (0.02)**	.**99 (<.01)**
Roseate tern	TerrOnly	0.76 (0.05)	0.92 (0.02)	.99 (<.01)
	MarOnly	0.81 (0.04)	0.94 (0.02)	.99 (<.01)
	**Terr w/Mar**	**0.93 (0.03** **)**	**0.98 (0.01)**	.**98 (<.01)**
	Mar w/Terr	0.88 (0.03)	0.96 (0.01)	.99 (<.01)

Note

Approach indicates the type of model run, TerrOnly represents “Terrestrial variables only”, MarOnly represents “Marine variables only”, Terr w/Mar represents “Terrestrial variables with the mean and standard deviation of nearby marine cells included,” and Mar w/Terr represents “Marine variables with the nearest terrestrial cell included.” True skill statistic (TSS) and receiving operator curve (ROC) indicate predictive accuracy, scaled between 0 and 1, where 1 indicates higher specificity and sensitivity. Mean (±SD) TSS and ROC values are averages taken from all internal cross‐validation models, excluding SDMs that were below the accuracy threshold (TSS < 0.6). Spearman's *p* is calculated between the ensemble projections made using 70% and 100% of presence/pseudo‐absence data sets and indicates whether the validation statistics are representative of the final model. An extended result table is available in Table S1. The highest‐performing model is highlighted in bold.

Agreement was high across ensemble models, with generally low uncertainty (Figures S2,S3). Ensemble models from different datasets and algorithms agreed most in the most suitable areas (around coastal areas), while uncertainty increased in more unsuitable areas (i.e., more inland areas). Since correlation between validation and full models was high, it seems unlikely that using all available data overfit models, and therefore, all tables and plots here that refer to “ensemble” models refer to those made with all available data.

**FIGURE 3:**
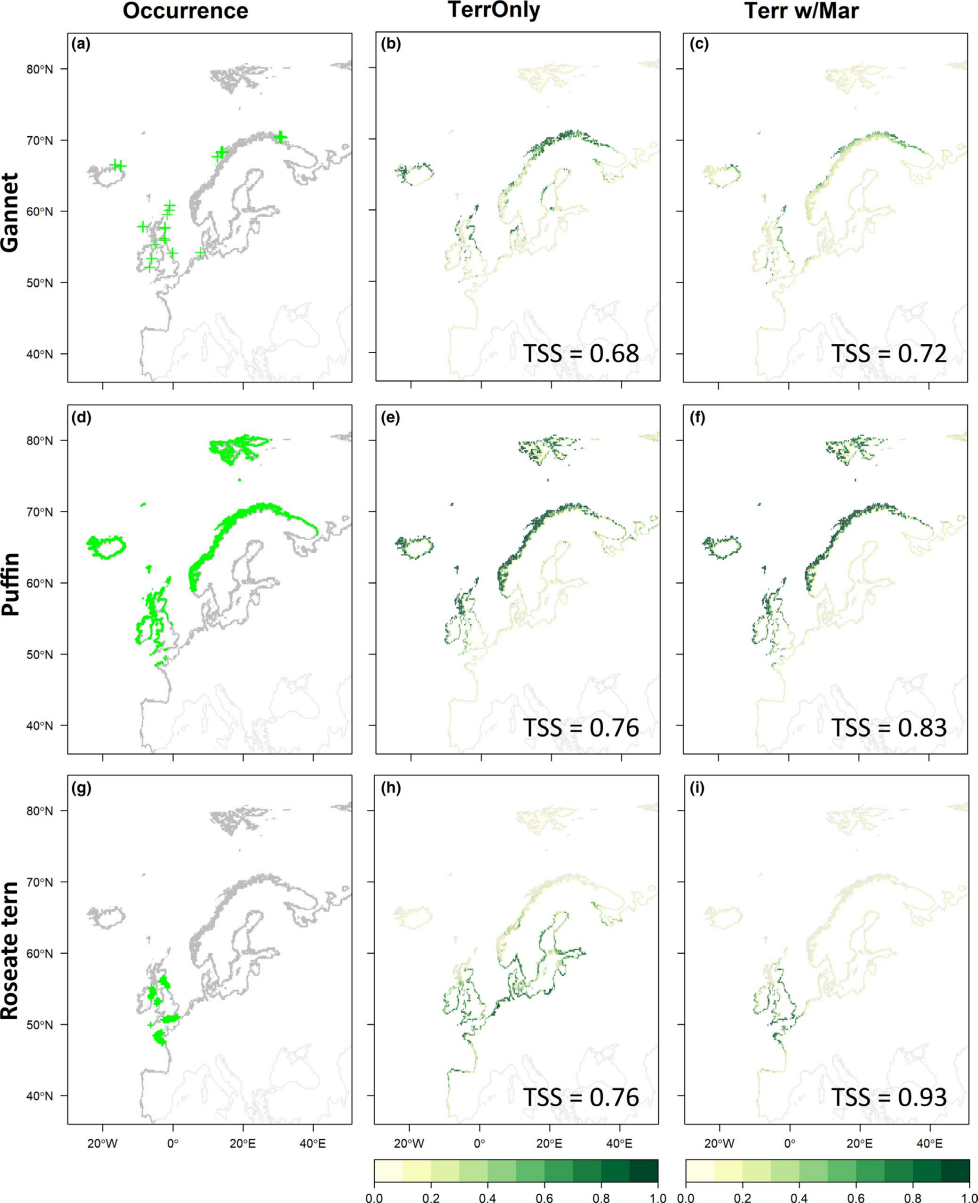
Terrestrial occurrence datasets for each species and output of single‐ and multi‐realm species distribution models (SDMs). (a, d, g) Terrestrial occurrences of northern gannets, Atlantic puffins, and roseate terns, respectively. (b, e, h) Ensemble SDM projections for each species built using terrestrial variables only. (c, f, i) Ensemble SDM projections for each species built using terrestrial variables with additional marine components. All ensemble models were made using all available distribution data and five pseudo‐absence datasets. Models were only included if their cross‐validation accuracy was above the threshold (true skill statistic > 0.6). Each ensemble was calculated as mean of projections from all included SDMs, weighted by the cross‐validated TSS

**FIGURE 4:**
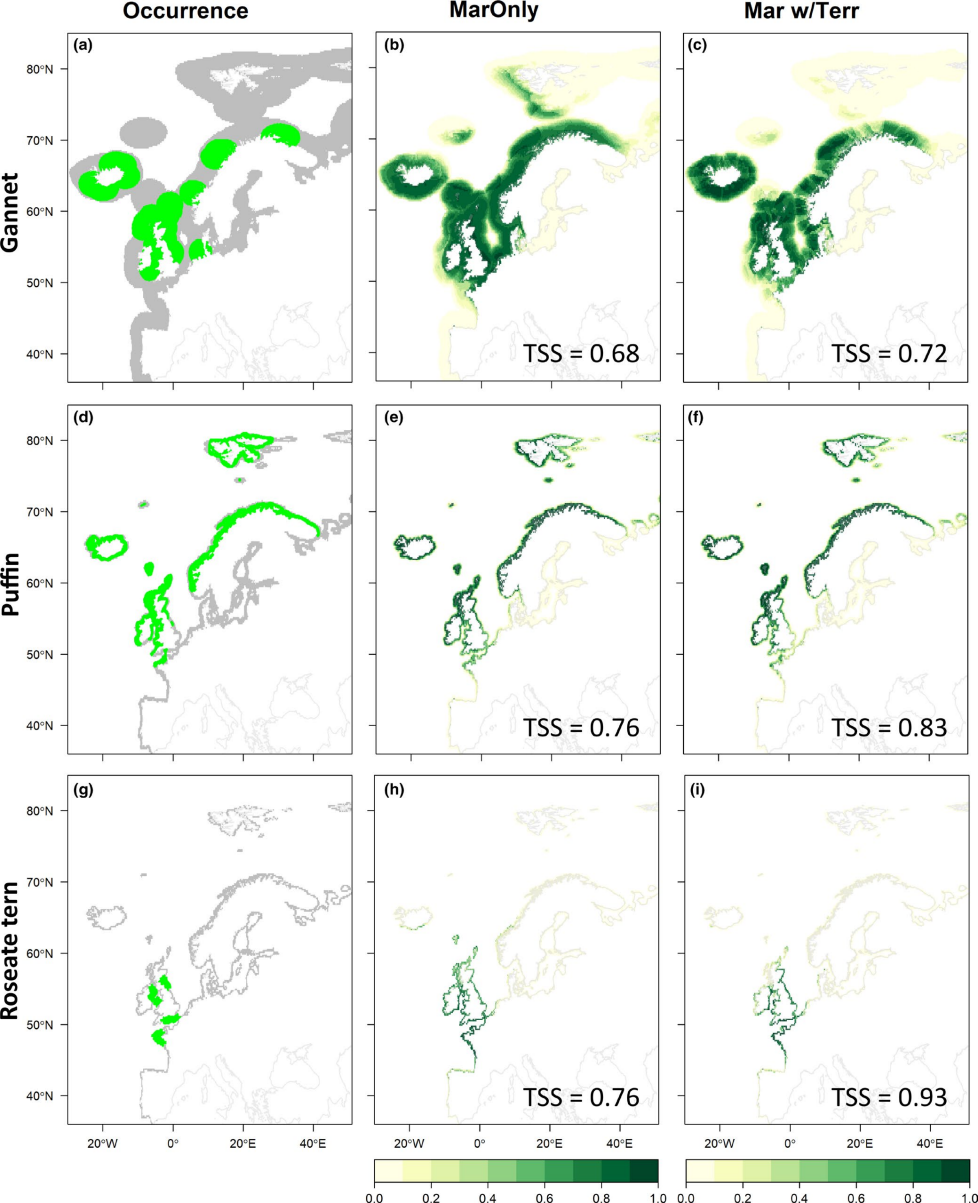
Marine occurrence datasets for each species and output of single‐ and multi‐realm species distribution models (SDMs). (a, d, g) Marine occurrences of Northern gannets, Atlantic puffins, and roseate terns, respectively. (b, e, h) Ensemble SDM projections for each species built using marine variables only. (c, f, i) Ensemble SDM projections for each species built using marine variables with additional terrestrial components. All ensemble models were made using all available distribution data and five pseudo‐absence datasets. Models were only included if their cross‐validation accuracy was above the threshold (true skill statistic > 0.6). Each ensemble was calculated as mean of projections from all included SDMs, weighted by the cross‐validated TSS

**FIGURE 5:**
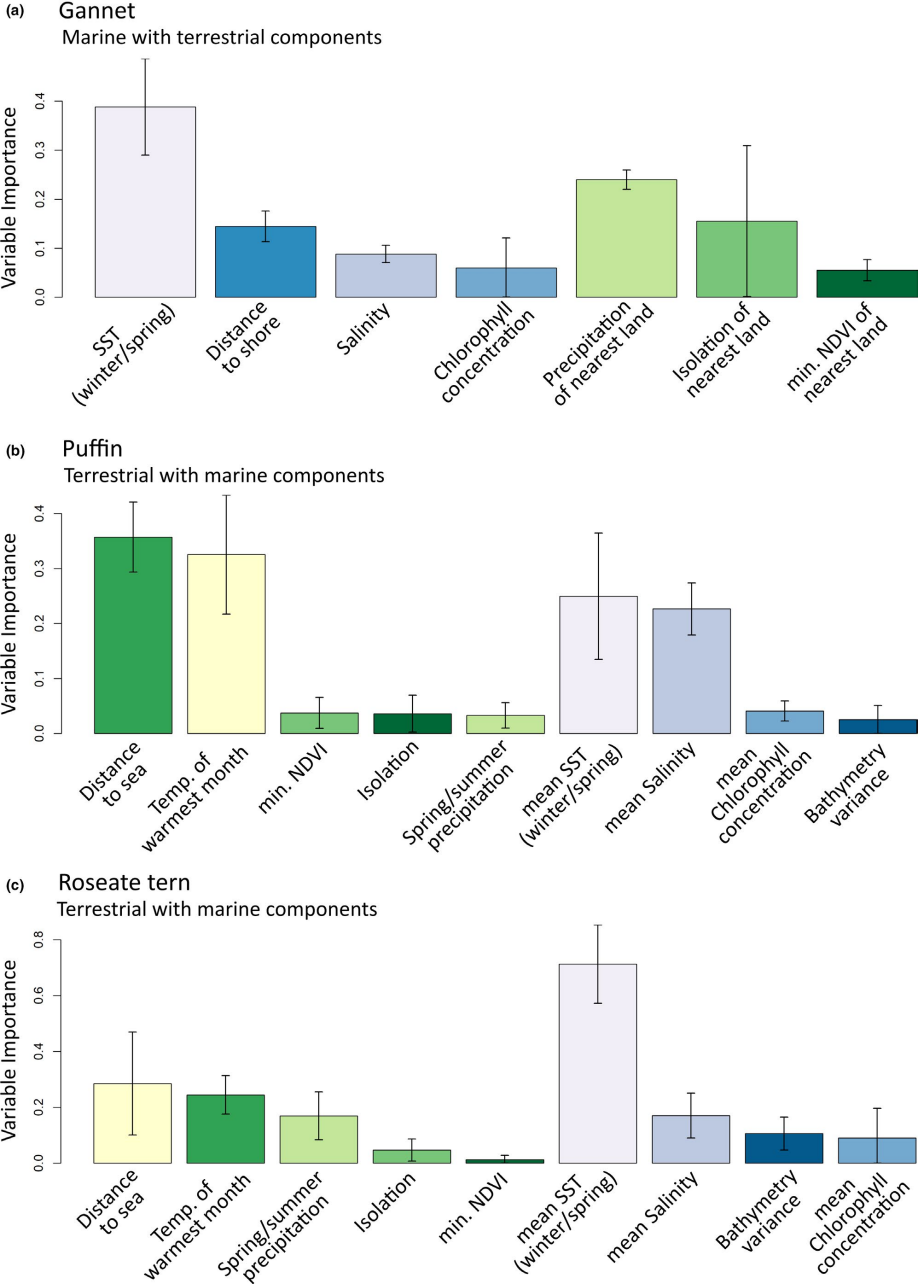
Variable importance for each species taken from the ensemble model with the highest true skill statistic score. Error bars are standard deviations of variable importance across all species distribution models. To see full model results for all four model types across the three species, see Table S2

For the three species considered, both terrestrial and marine models were improved by the addition of marine and terrestrial variables, respectively (Table 1; Figure 3; Figure 4). For the Atlantic puffin, the best modeling approach was a terrestrial combined with marine information from the surrounding area (TSS = 0.76; ROC = 0.95; Table 1). The most important terrestrial variables for this species were mean temperature of the warmest month (Pearson's *r* = .33) and distance from the sea (Pearson's *r* = .36); the most important marine variables were mean SST in nearby marine areas during the winter/spring (Pearson's *r* = .25) and the mean salinity of surrounding areas (Pearson's *r* = .23). Isolation, minimum NDVI, precipitation during the spring and summer, chlorophyll concentration, and bathymetry variance were retained also in the final model, but had low overall importance (Pearson's *r* < .05; Figure 5; Table S2).

For the Northern gannet, the best modeling approach was a marine model with terrestrial components (TSS = 0.75; ROC = 0.93; Table 1). The most important terrestrial variables for this species were spring and summer precipitation on the nearest land (Pearson's *r* = .24), isolation of the nearest land (Pearson's *r* = .16), and mean NDVI of the nearest land (Pearson's *r* = .06; Figure 5); the most important marine variables were SST during the winter and spring (Pearson's *r* = .39), distance to shore (Pearson's *r* = .14), mean salinity (Pearson's *r* = .09), and maximum chlorophyll concentration (Pearson's *r* = .06; see also Figure 5; Table S2).

For roseate terns, the best modeling approach was a terrestrial model with additional marine components (TSS = 0.93; ROC = 0.98; Table 1). The most important terrestrial variables were mean temperature of warmest month (Pearson's *r* = .29), distance to the sea (Pearson's *r* = .24), and precipitation during the breeding season (Pearson's *r* = .17); the most important marine variables for this species were SST during the winter and spring (Pearson's *r* = .71), mean salinity (Pearson's *r* = .17), and the variability of bathymetry (Pearson's *r* = .11). Isolation of landmass and minimum NDVI were also retained in the final model but had low overall importance (Pearson's *r* = <.05; Figure 5; Table S2).

## 4 DISCUSSION

A general principle of SDMs is that any ecologically relevant variable that could strongly influence a species range should be included. If a species has both a marine range and a terrestrial range, and both terrestrial and marine variables are important, the next logical step is to include both realms in a single model framework. However, existing SDMs typically do not effectively combine information from multiple realms, even when considering species that utilize multiple realms, such as seals, sea turtles, sea snakes, and seabirds. Previous work, particularly in seabirds, has typically either selected marine or terrestrial variables as the main realm and used variables exclusively from one or the other (Bosch et al., 2018; Quillfeldt et al., 2017; Waggitt et al., 2020). In contrast to this, we combined terrestrial and marine information by sampling from the marine foraging range (in the case of terrestrial models) or sampling from the nearest coastal region (in the case of marine models). Our results demonstrate that the addition of marine variables to terrestrial SDMs, or vice versa, improves the accuracy of SDMs for multi‐realm species, in both specificity and sensitivity. Through a combination of internal validation and pseudo‐absence selection, we found that models were overall robust, with little sensitivity to loss of data in model training; there was, moreover, little evidence of over‐fitting. However, the degree of improvement and the best method for combining marine and terrestrial factors varied between species. Our study thus demonstrates that this approach provides a general framework for combining terrestrial and marine variables in a single SDM, in a repeatable and transferable way.

The three species we considered have different ecologies, which was reflected in the different patterns captured by our SDMs. Perhaps the most striking pattern was that, although multi‐realm SDMs systematically performed better than single realm ones, the importance of particular environmental variables varied greatly, as did the extent to which the marine or terrestrial factors dominated. Previous work on seabird ranges has demonstrated the importance of marine variables (Bosch et al., 2018; Engler et al., 2017), but interestingly, we found evidence to suggest that some seabird breeding ranges are more strongly determined by terrestrial components than by marine ones. For example, we found that models with terrestrial variables were more accurate for puffins than models without these variables and that terrestrial temperature during the summer was at least as important as sea surface temperature during the winter. This is somewhat surprising as winter marine temperature is closely correlated to marine productivity overall (Engler et al., 2017; Huettmann et al., 2011; Quillfeldt et al., 2017). Since puffins have strict preferences for marine foraging areas (BirdLife International, 2021a), we would expect its range to be more closely correlated to marine conditions than terrestrial ones. From this form of correlative SDM, it is not possible to unravel the mechanism by which terrestrial temperature of the hottest month affects puffins, but our results suggest that to understand how puffin ranges are structured and how climate change may affect them, then the role of terrestrial temperature should be investigated with some urgency. In that respect, future studies could consider the mechanism of how rising terrestrial temperatures could affect puffins, for example, whether high temperatures in the burrow negatively affect chicks and under what parameters, or whether high air temperatures prevent adults from dissipating heat generated while foraging effectively (Oswald et al., 2021; Schraft et al., 2019). By contrast, marine variables were much more important than terrestrial ones for roseate terns. While multi‐realm models performed better overall, winter/spring SST was found to be the most important variable in all models in which it was included, and by a significant margin. This is another example where a species range is affected by the conditions of one realm more than another, and has potential consequences for future modeling and conservation planning. Since roseate terns have a broad tolerance for terrestrial temperatures and habitat (BirdLife International, 2021c), changes at sea are thus much more likely to impact their populations.

Notably, our work highlights the importance of the manner and the order in which information on realms is combined in SDMs, with the best method for combining marine and terrestrial factors differing among the species considered. For example, a terrestrial model with marine components performed well overall for puffins and established high importance of terrestrial temperature, sea temperature, and salinity. The inverse model to this, a marine model with terrestrial factors, found the same factors to be important (Table S2), but not with the same accuracy (Table 1). This indicates that, at least in this case, not all methods of combining marine and terrestrial information are equal and that care should be taken when considering how to combine information.

However, adding multi‐realm information does not always result in dramatic model improvement, and there are several possible ways to improve model accuracy and confidence with further development. In particular, care should be taken in interpreting how and where breeding localities are predicted; marine models with terrestrial information only consider land close to the coast and are therefore limited for predicting breeding sites further inland. In addition, presence distributions are inherently limited by the location of breeding localities within the models and currently cannot be applied away from breeding localities. There are likely a number of other variables that could greatly influence seabird ranges but that were not included because either dataset was not available or they were difficult to incorporate in our framework. For example, many seabird species, such as gannets, are highly colonial and may select colonies based on fine scale topographical and geomorphological features, in particular the aspect, slope, size, and orientation of steep rocky cliffs (BirdLife International, 2021b) or on the distance to other colonies (Grecian et al., 2012; Lewis et al., 2001). Using high‐resolution occurrence and environmental data to model seabird ranges may reveal further insights into what factors are most important to the placement and success of breeding colonies, and therefore how breeding ranges may change in the future. There are in addition numerous non‐environmental factors that may influence seabird ranges and may explain why some models overestimated species ranges (Estrada et al., 2017; Gaston, 2009). In particular, high mortality caused by introduced predators, by‐catch, and human persecution of seabirds have caused several recent and historical extinctions, sometimes followed by recolonization (Dias et al., 2019), and are likely to be strong drivers of seabird range change in the future along with climate. Many seabird ranges are moreover greatly influenced by the distribution of their prey species, and many of the marine variables we used, such as SST and chlorophyll concentration, are proxies to describe prey ranges. These variables are commonly used because fish species ranges are often poorly described, especially for a cryptic species of low commercial interest (Grecian et al., 2012). The marine variables in our models are proxy variables and are mostly unlikely to affect seabird species themselves directly (Engler et al., 2017). Including more accurate distribution information on prey species such as sandeels may show that marine variables have a stronger effect on seabird ranges than we found here.

To conserve species effectively, we need ways to understand and model their current ranges and how they may change in the future. For species that span the marine and terrestrial realms, this includes understanding the relative importance of marine and terrestrial realms and the relationship between them. Our approach has a few advantages over existing alternative methods, by combining terrestrial and marine factors into a single model. Overall, the method is relatively simple and uses standard SDM approaches to identify overall suitability across the area of interest. Our approach does not require post hoc combination or the use of thresholds on several different models and therefore is less computationally expensive than existing approaches available to model habitat suitability of species dependent on more than one ecological realm. For each species, a maximum distance parameter is needed to summarize effectively relevant marine information around a terrestrial point and vice versa; we used mean maximum foraging distance (MMFD). Such parameters are well recorded for many seabirds due to the availability of tracking data, and we believe our approach is transposable to other species, as long as a sensible “foraging” distance is known, or an alternative parameter can be identified. There are further applications for a multi‐realm approach, either through further development or from combination with other techniques. In particular, as our approach includes terrestrial and marine variables in a single model, it can reveal novel relationships and interactions between different variables; for example, we found that Atlantic puffins’ ranges are strongly structured by marine and terrestrial temperatures but in different ways for different seasons. Anthropogenic climate change is likely to result in wide‐scale changes in both the marine and terrestrial realms, and multi‐realm models can assess how it may impact not just species in each realm, but also, crucially, how these changes may interact to impact multi‐realm species across both their terrestrial and marine ranges. Given their overall high sensitivity and specificity, improved performance and transferability to many different species, we believe multi‐realm SDMs are a valuable tool to aid researchers and practitioner's understanding of what structures multi‐realm species’ ranges, helping identify new patterns and relationships in species’ ranges, and to improve predictions of suitability in the future.

## CONFLICT OF INTEREST

The authors declare no conflict of interest.

## AUTHOR CONTRIBUTION

**Henry Hakkinen:** Conceptualization (equal); Data curation (lead); Formal analysis (lead); Investigation (equal); Methodology (lead); Software (lead); Visualization (lead); Writing‐original draft (lead); Writing‐review & editing (equal). **Silviu O. Petrovan:** Conceptualization (equal); Funding acquisition (equal); Investigation (equal); Methodology (supporting); Writing‐review & editing (equal). **William J. Sutherland:** Conceptualization (equal); Funding acquisition (equal); Investigation (equal); Methodology (supporting); Writing‐review & editing (equal). **Nathalie Pettorelli:** Conceptualization (equal); Funding acquisition (equal); Investigation (equal); Methodology (supporting); Supervision (lead); Writing‐original draft (supporting); Writing‐review & editing (equal).

## Supporting information

Fig S1

Fig S2

Fig S3

Table S1‐S2

## Data Availability

The underlying code and data can be accessed at https://doi.org/10.5281/zenodo.5565397 (https://doi.org/10.5281/zenodo.5565397). Links and details to acquire any additional data required are also included.
