# Supplementary figures and images for: Terrestrial or marine species distribution model: Why not both? A case study with seabirds
Source: Ecol Evol. 2021 Nov 23;11(23):16634–46. doi: 10.1002/ece3.8272 (PMC8668722; doi:10.1002/ece3.8272)

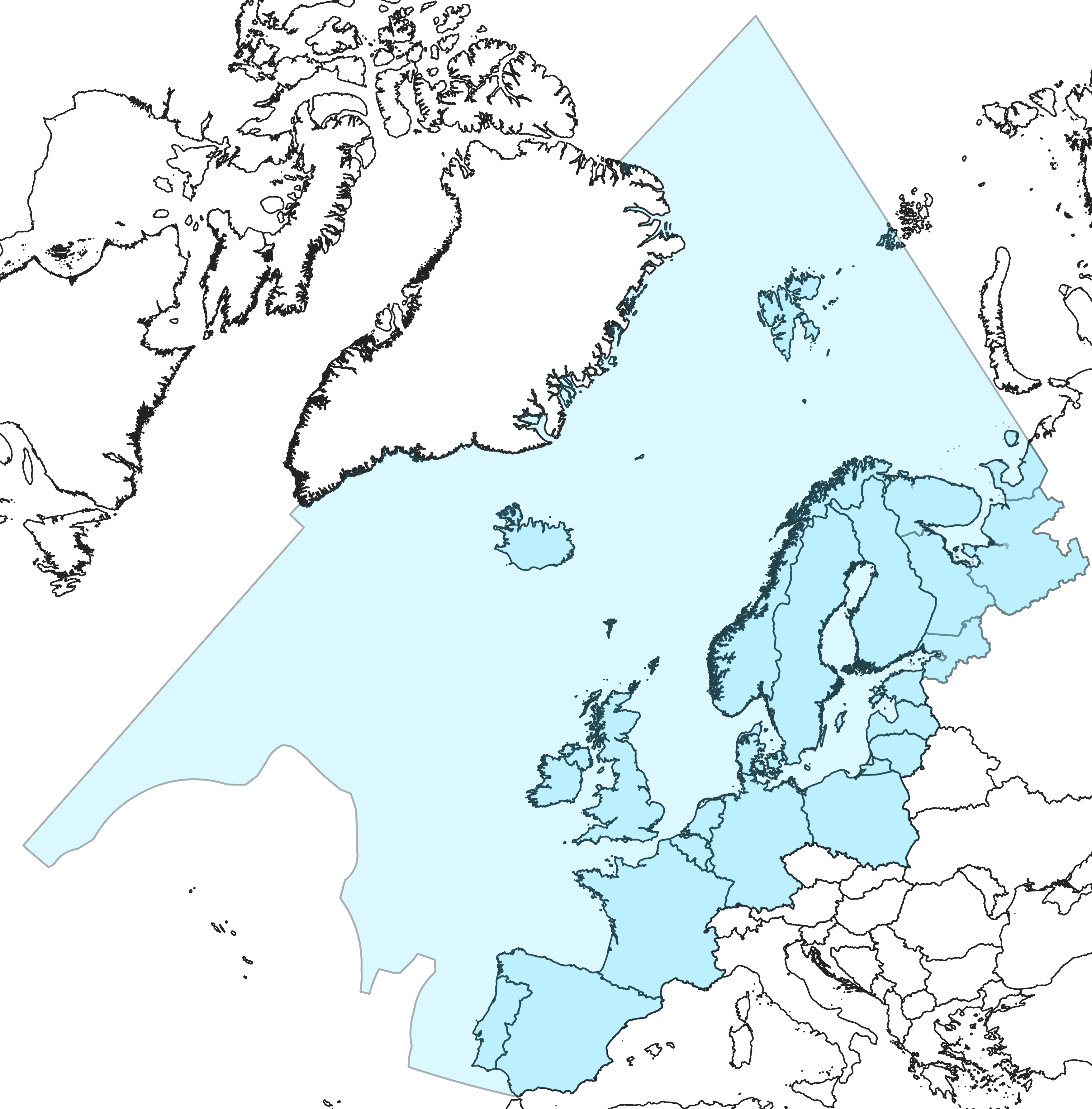

Supplement: Supplementary file 1 — Fig S1 [file ECE3-11-16634-s003.tif]

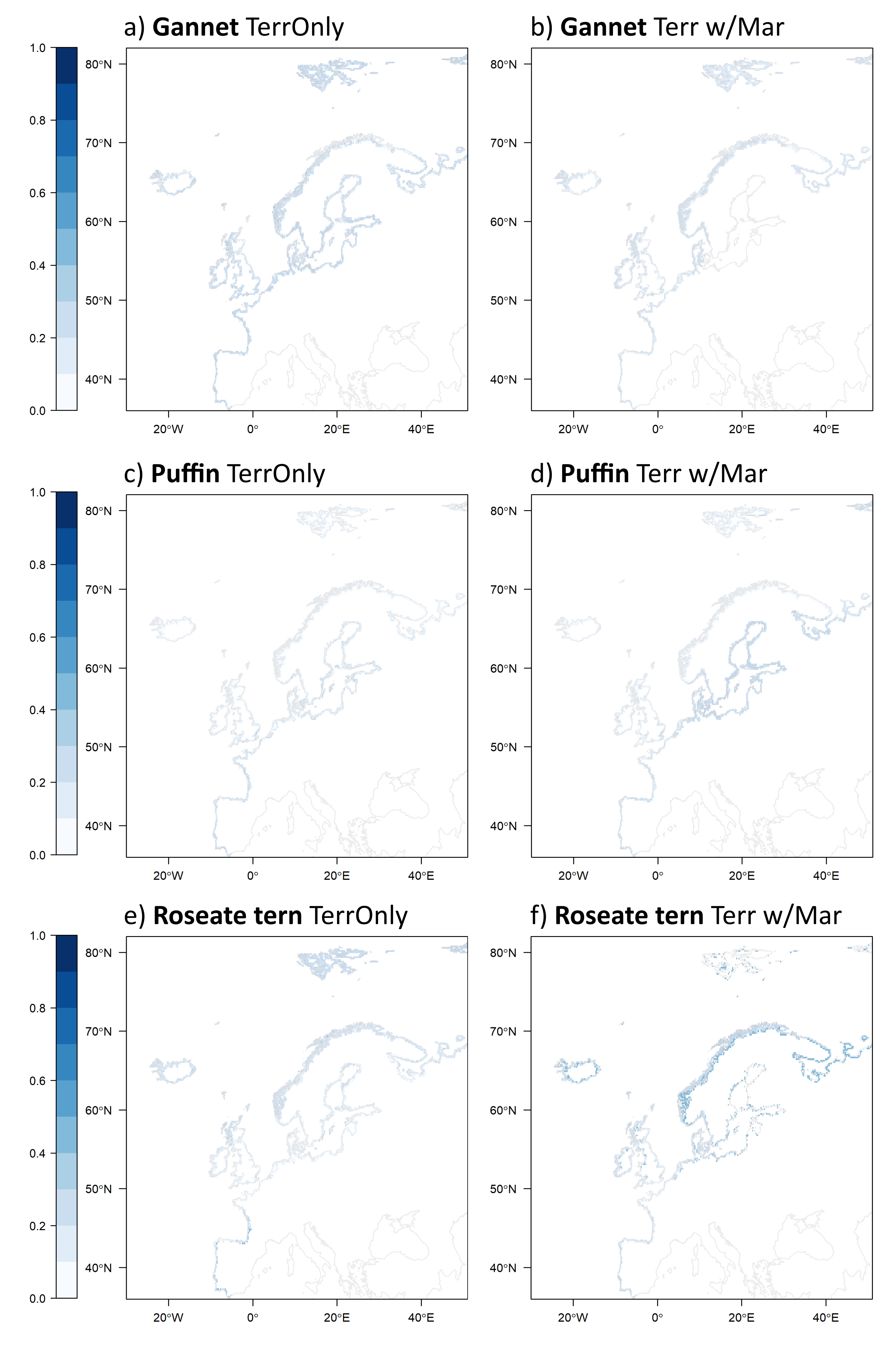

Supplement: Supplementary file 2 — Fig S2 [file ECE3-11-16634-s001.tif]

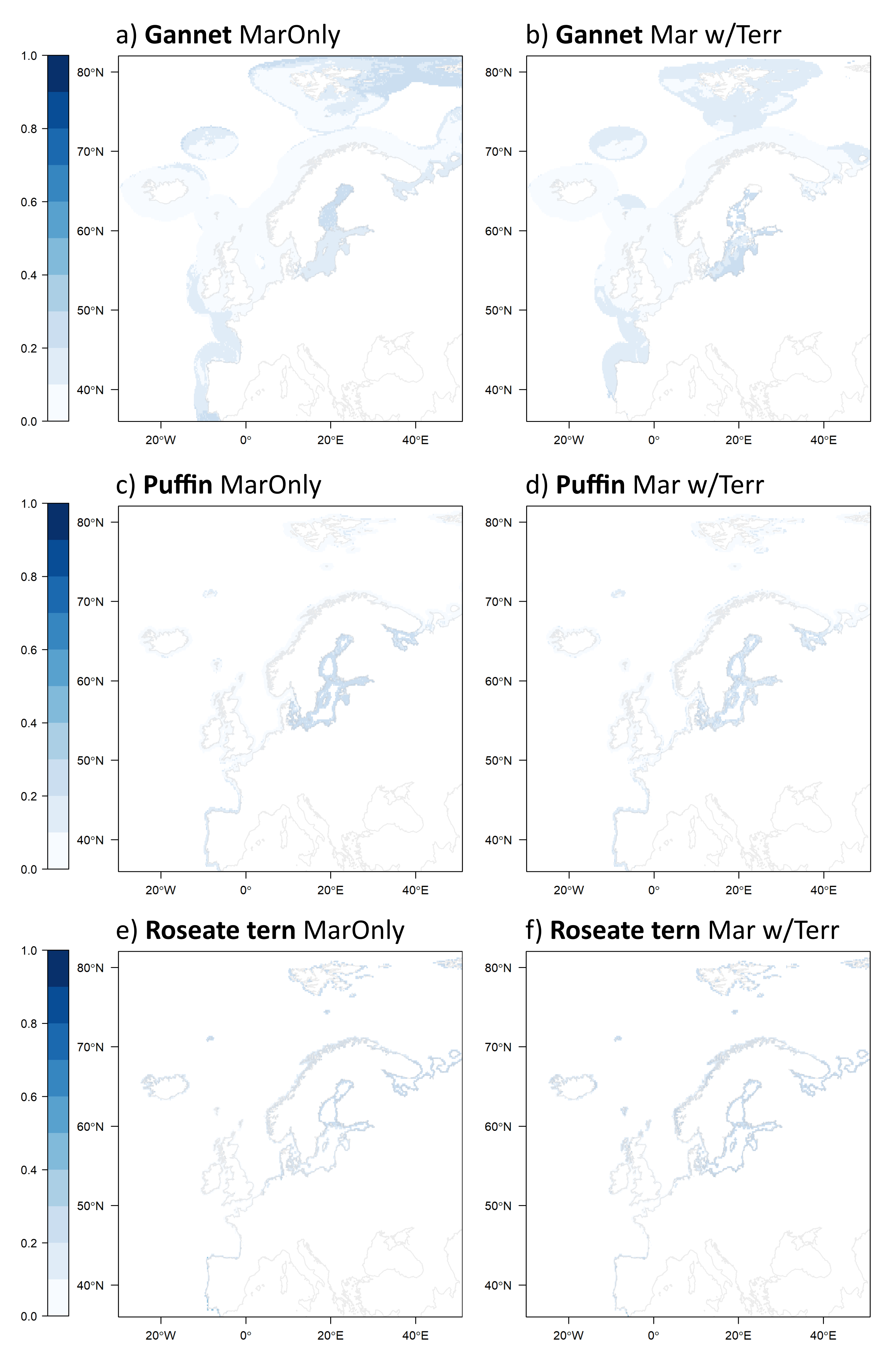

Supplement: Supplementary file 3 — Fig S3 [file ECE3-11-16634-s004.tif]
