# Supplementary material for: Terrestrial or marine species distribution model: Why not both? A case study with seabirds
Source: Ecol Evol. 2021 Nov 23;11(23):16634–46. doi: 10.1002/ece3.8272 (PMC8668722; doi:10.1002/ece3.8272)
Supplement: Supplementary file 4 — Table S1‐S2 [file ECE3-11-16634-s002.docx]

**Appendix**

**Table S1:** Summary statistics for Species Distribution Models (SDMs) for each species and modelling approach. Approach indicates the type of model run, TerrOnly represents “Terrestrial variables only”, MarOnly represents “Marine variables only”, Terr w/Mar represents “Terrestrial variables with the mean and standard deviation of nearby marine cells included” and Mar w/Terr represents “Marine variables with the nearest terrestrial cell included”. True Skill Statistic (TSS) and Receiving Operator Curve (ROC) indicate predictive accuracy, scaled between 0 and 1, where 1 indicates higher specificity and sensitivity. Sensitivity, specificity and accuracy are the true positive rate, the true negative rate and correct assessment rate respectively. Spearman’s p is calculated between the ensemble projections made using 70% and 100% of presence/pseudoabsence data sets, and indicate whether the validation statistics are representative of the final model. All values not in brackets are the mean value from all internal cross-validation models, excluding SDMs that were below the accuracy threshold (TSS<0.6). Figures in brackets are the standard deviation from the same set of models as for the mean.

| Species | Approach | TSS | ROC | SENSITIVITY | SPECIFICITY | Accuracy | Spearman’s p |
| --- | --- | --- | --- | --- | --- | --- | --- |
| Atlantic puffin | TerrOnly | 0.76 (0.02) | 0.95 (0.01) | 0.88 (0.09) | 0.78 (0.18) | 0.99 (<0.01) | 0.99 (<0.01) |
|  | MarOnly | 0.65 (0.03) | 0.91 (0.01) | 0.911 (0.12) | 0.80 (0.09) | 0.91 (0.03) | 0.99 (<0.01) |
|  | **Terr w/Mar** | **0.83 (0.02)** | **0.97 (<0.01)** | **0.88 (0.08)** | **0.84 (0.13)** | **0.99 (<0.01)** | **0.99 (<0.01)** |
|  | Mar w/Terr | 0.69 (0.03) | 0.93 (0.01) | 0.93 (0.02) | 0.82 (0.03) | 0.93 (0.01) | 0.99 (<0.01) |
| Northern gannet | TerrOnly | 0.68 (0.10) | 0.87 (0.05) | 0.88 (0.03) | 0.90 (0.02) | 0.89 (0.02) | 0.74 (0.17) |
|  | MarOnly | 0.69 (0.04) | 0.89 (0.02) | 0.88 (0.04) | 0.83 (0.05) | 0.86 (0.04) | 0.99 (<0.01) |
|  | Terr w/Mar | 0.72 (0.13) | 0.88 (0.07) | 0.9271 (0.03) | 0.92 (0.02) | 0.92 (0.02) | 0.93 (0.02) |
|  | **Mar w/Terr** | **0.75 (0.04)** | **0.93 (0.02)** | **0.89 (0.050** | **0.85 (0.05)** | **0.88 (0.05)** | **0.99 (<0.01)** |
| Roseate tern | TerrOnly | 0.76 (0.05) | 0.92 (0.02) | 0.93 (0.03) | 0.83 (0.04) | 0.96 (<0.01) | 0.99 (<0.01) |
|  | MarOnly | 0.81 (0.04) | 0.94 (0.02) | 0.98 (0.01) | 0.86 (0.06) | 0.94 (0.02) | 0.99 (<0.01) |
|  | **Terr w/Mar** | **0.93 (0.03)** | **0.98 (0.01)** | **0.98 (0.02)** | **0.9421 (0.03)** | **0.98 (0.01)** | **0.98 (<0.01)** |
|  | Mar w/Terr | 0.88 (0.03) | 0.96 (0.01) | 0.98 (0.01) | 0.91 (0.02) | 0.95 (0.02) | 0.99 (<0.01) |

**Table S2:** Summary of variable importance across all species distribution models (SDMs). For each species, and each model type we assessed the relative importance of each variable in predicting presences and absences in our dataset. Importance is scaled from 0 to 1, where 0 indicates a given variable has no importance on predicting the presence of the species, and 1 indicates a variable can be used to completely predict the presence of the species. Approach indicates the type of model run, TerrOnly represents “Terrestrial variables only”, MarOnly represents “Marine variables only”, Terr/wMar represents “Terrestrial variables with the mean and standard deviation of nearby marine cells included” and Mar w/Terr represents “Marine variables with the nearest terrestrial cell included”. Values given are the mean importance across all models and the lower and upper confidence interval (CI).

| Species | Approach | Variable | Mean | upper CI | lower CI |
| --- | --- | --- | --- | --- | --- |
| Atlantic puffin | Terr Only | Temp of warmest month | 0.53 | 0.59 | 0.47 |
|  |  | Distance to sea | 0.44 | 0.49 | 0.38 |
|  |  | Spr/Sum precip | 0.21 | 0.28 | 0.15 |
|  |  | Isolation | 0.06 | 0.11 | 0.01 |
|  |  | Minimum NDVI | 0.04 | 0.07 | 0 |
|  | Terr w/Marine | Distance to sea | 0.36 | 0.42 | 0.29 |
|  |  | Temp of warmest month | 0.33 | 0.43 | 0.22 |
|  |  | Isolation | 0.04 | 0.07 | 0.01 |
|  |  | Minimum NDVI | 0.04 | 0.07 | 0 |
|  |  | Spr/Sum precip | 0.03 | 0.06 | 0.01 |
|  |  | Mean Win/Spr SST | 0.25 | 0.36 | 0.13 |
|  |  | Mean salinity | 0.23 | 0.27 | 0.18 |
|  |  | Mean chlorophyll | 0.04 | 0.06 | 0.02 |
|  |  | Bathymetry var | 0.03 | 0.05 | 0 |
|  | Marine Only | Mean Salinity | 0.62 | 0.67 | 0.58 |
|  |  | Distance to shore | 0.25 | 0.3 | 0.2 |
|  |  | SST (Winter/Spring) | 0.24 | 0.29 | 0.18 |
|  |  | Max. chlorophyll | 0.15 | 0.21 | 0.09 |
|  | Marine w/Terr | Mean Salinity | 0.49 | 0.57 | 0.41 |
|  |  | SST (Winter/Spring) | 0.23 | 0.28 | 0.18 |
|  |  | Distance to shore | 0.22 | 0.28 | 0.16 |
|  |  | Max. chlorophyll | 0.14 | 0.19 | 0.09 |
|  |  | Spr/Sum precip on nearest land | 0.16 | 0.23 | 0.08 |
|  |  | Isolation of nearest land | 0.06 | 0.1 | 0.02 |
|  |  | NDVI on nearest land | 0.02 | 0.04 | 0 |
| Northern gannet | Terr Only | Temp of warmest month | 0.57 | 0.81 | 0.33 |
|  |  | Spr/Sum precip | 0.38 | 0.68 | 0.08 |
|  |  | Distance to sea | 0.37 | 0.58 | 0.16 |
|  |  | Isolation | 0.22 | 0.43 | 0 |
|  |  | Minimum NDVI | 0.1 | 0.18 | 0.02 |
|  | Terr w/Marine | Temp of warmest month | 0.57 | 0.82 | 0.33 |
|  |  | Spr/Sum precip | 0.33 | 0.58 | 0.07 |
|  |  | Isolation | 0.25 | 0.32 | 0.17 |
|  |  | Distance to sea | 0.17 | 0.18 | 0.15 |
|  |  | Minimum NDVI | 0.09 | 0.21 | 0 |
|  |  | Mean salinity | 0.59 | 0.7 | 0.47 |
|  |  | Mean Win/Spr SST | 0.55 | 0.77 | 0.32 |
|  |  | Mean chlorophyll | 0.53 | 0.78 | 0.29 |
|  |  | Bathymetry var | 0.39 | 0.7 | 0.08 |
|  | Marine Only | SST (Winter/Spring) | 0.72 | 0.75 | 0.68 |
|  |  | Distance to shore | 0.24 | 0.3 | 0.18 |
|  |  | Mean Salinity | 0.14 | 0.21 | 0.07 |
|  |  | Max. chlorophyll | 0.1 | 0.19 | 0.01 |
|  | Marine w/Terr | SST (Winter/Spring) | 0.39 | 0.49 | 0.29 |
|  |  | Distance to shore | 0.14 | 0.18 | 0.11 |
|  |  | Mean Salinity | 0.09 | 0.11 | 0.07 |
|  |  | Max. chlorophyll | 0.06 | 0.12 | 0 |
|  |  | Spr/Sum precip on nearest land | 0.24 | 0.26 | 0.22 |
|  |  | Isolation of nearest land | 0.16 | 0.31 | 0 |
|  |  | NDVI on nearest land | 0.06 | 0.08 | 0.03 |
| Roseate tern | Terr Only | Temp of warmest month | 0.58 | 0.67 | 0.5 |
|  |  | Distance to sea | 0.51 | 0.59 | 0.42 |
|  |  | Spr/Sum precip | 0.36 | 0.56 | 0.16 |
|  |  | Isolation | 0.09 | 0.14 | 0.04 |
|  |  | Minimum NDVI | 0.05 | 0.11 | 0 |
|  | Terr w/Marine | Temp of warmest month | 0.29 | 0.47 | 0.1 |
|  |  | Distance to sea | 0.24 | 0.31 | 0.18 |
|  |  | Spr/Sum precip | 0.17 | 0.26 | 0.08 |
|  |  | Isolation | 0.05 | 0.09 | 0.01 |
|  |  | Minimum NDVI | 0.01 | 0.03 | 0 |
|  |  | Mean Win/Spr SST | 0.71 | 0.85 | 0.57 |
|  |  | Mean salinity | 0.17 | 0.25 | 0.09 |
|  |  | Bathymetry var | 0.11 | 0.17 | 0.05 |
|  |  | Mean chlorophyll | 0.09 | 0.2 | 0 |
|  | Marine Only | SST (Winter/Spring) | 0.87 | 0.92 | 0.83 |
|  |  | Mean Salinity | 0.26 | 0.41 | 0.11 |
|  |  | Max. chlorophyll | 0.1 | 0.16 | 0.04 |
|  |  | Distance to shore | 0.02 | 0.05 | 0 |
|  | Marine w/Terr | SST (Winter/Spring) | 0.72 | 0.85 | 0.59 |
|  |  | Mean Salinity | 0.2 | 0.28 | 0.13 |
|  |  | Max. chlorophyll | 0.04 | 0.07 | 0.01 |
|  |  | Distance to shore | 0.01 | 0.03 | 0 |
|  |  | Spr/Sum precip on nearest land | 0.28 | 0.35 | 0.21 |
|  |  | Isolation of nearest land | 0.08 | 0.15 | 0.02 |
|  |  | NDVI on nearest land | 0.02 | 0.03 | 0.01 |
